# Effect of Material Composition on Tunable Surface Roughness of Magnetoactive Elastomers

**DOI:** 10.3390/polym11040594

**Published:** 2019-04-01

**Authors:** Gašper Glavan, Wolfgang Kettl, Alexander Brunhuber, Mikhail Shamonin, Irena Drevenšek-Olenik

**Affiliations:** 1 East Bavarian Centre for Intelligent Materials (EBACIM), Ostbayerische Technische Hochschule (OTH) Regensburg, Seybothstr. 2, D-93053 Regensburg, Germany; wolfgangkettl@rocketmail.com (W.K.); a.brunhuber@hotmail.com (A.B.); mikhail.chamonine@oth-regensburg.de (M.S.); 2 Faculty of Mathematics and Physics, University of Ljubljana, Jadranska 19, SI1000 Ljubljana, Slovenia; irena.drevensek@ijs.si; 3 J. Stefan Institute, Jamova 39, SI1000 Ljubljana, Slovenia

**Keywords:** magnetorheological polymers, surface properties, magnetically tunable surface roughness, magnetically tunable surface reflectivity

## Abstract

We investigated magnetic-field-induced modifications of the surface roughness of magnetoactive elastomers (MAEs) with four material compositions incorporating two concentrations of ferromagnetic microparticles (70 wt% and 80 wt%) and exhibiting two shear storage moduli of the resulting composite material (about 10 kPa and 30 kPa). The analysis was primarily based on spread optical reflection measurements. The surfaces of all four materials were found to be very smooth in the absence of magnetic field (RMS roughness below 50 nm). A maximal field-induced roughness modification (approximately 1 μm/T) was observed for the softer material with the lower filler concentration, and a minimal modification (less than 50 nm/T) was observed for the harder material with the higher filler concentration. All four materials showed a significant decrease in the total optical reflectivity with an increasing magnetic field as well. This effect is attributed to the existence of a distinct surface layer that is depleted of microparticles in the absence of a magnetic field but becomes filled with particles in the presence of the field. We analyzed the temporal response of the reflective properties to the switching on and off of the magnetic field and found switching-on response times of around 0.1 s and switching-off response times in the range of 0.3–0.6 s. These observations provide new insight into the magnetic-field-induced surface restructuring of MAEs and may be useful for the development of magnetically reconfigurable elastomeric optical surfaces.

## 1. Introduction

Magnetoactive elastomers (MAEs), also known as magnetorheological elastomers (MREs) in the context of mechanical applications, are smart materials composed of micrometer-sized ferromagnetic particles embedded in a soft polymer matrix [1,2,3,4,5,6,7,8,9,10,11]. They can be considered solid analogies of magnetorheological fluids [12,13]. Matrix softness is one of the most essential properties of MAEs, as it facilitates a large spatial rearrangement of filler particles even in relatively moderate magnetic fields (100–300 mT) [14]. This leads to significant magnetic-field-induced modifications of various physical properties, such as dynamic elastic moduli or electric properties [5,6,15,16,17,18,19,20,21,22,23,24,25,26,27]. The related bulk properties are quite well-investigated, although the physical processes behind the observed phenomena are not yet completely clarified and remain a subject of lively theoretical investigations; see, for example, References [7,10,28,29,30,31].

Very recently, the surface properties of MAEs have attracted the attention of several research groups, as it was demonstrated that they can be strongly modulated by applied magnetic fields as well [32,33,34,35,36,37,38,39,40,41]. This interest is driven by a large number of potential applications, ranging from magnetically controllable hydrophobic coatings [33] and biomedical substrates [42] to magnetically reconfigurable diffractive optical elements [34]. Magnetorheological fluids have been previously employed in a prototype tactile display [43]. Obviously, filler particles in the vicinity of the MAE-air interface are exposed to different interactions than particles in the interior of MAE. Nevertheless, it is reasonable to expect that some field-induced restructuring should be noticeable also on the surface. In our previous work, we investigated magnetic-field-induced modifications of the surface topography of a single MAE material with some specific composition. In this work, which is a follow-up of Reference [34], we investigate different materials and address the question of how their surface restructuring depends on the elastic properties and on the concentration of the ferromagnetic filler.

## 2. Materials and Methods

### 2.1. MAE Specimens

The polymer matrix of the investigated MAEs is made of the base polymer VS100000 (vinyl-functional polydimethylsiloxane (PDMS)) for addition-curing silicones and the reactive diluent polymer MV 2000 (monovinyl functional PDMS) combined with the chain extender modifier 715 (SiH-terminated PDMS; all these materials were provided by Evonik Hanse GmbH, Geesthacht, Germany) and silicone oil AK10 (linear, nonreactive PDMS manufactured by Wacker Chemie AG, Burghausen, Germany). To these initial ingredients, a carbonyl iron powder (CIP, type SQ, median diameter value of 3.9–5.0 μm, BASF SE Carbonyl Iron Powder & Metal Systems, Ludwigshafen, Germany), a cross linker 210 (dimethyl siloxane-methyl hydrogen siloxane copolymer), an inhibitor DVS and a Pt-catalyst 510 (all from Evonik Hanse GmbH, Geesthacht, Germany) are added. The purpose of different chemical reagents is explained elsewhere [44,45,46]. The hydrosilylation reaction is carried out in presence of the Pt (Karstedt’s) catalyst [47]. The PDMS polymerization mechanism is given, for example, in Scheme 1 of Reference [48].

The usage of silicone oil is essential for the convenient processing of elastomer composites because it allows one to keep the low viscosity of the initial colloidal mixture before commencing the curing process. The resulting liquid mixture is poured into a petri dish and placed into a vacuum chamber for about 10 min to remove air bubbles. After this, it is moved to an oven with air circulation, where it undergoes a curing process that takes place at first for one hour at 80 °C and then for 24 h at 60 °C. This happens in the absence of a magnetic field, so the incorporated microparticles should be randomly dispersed in the polymer matrix without any sign of order (chain-like structures, etc.). The samples in the form of disc-shaped plates with a diameter of 20 mm and a thickness of about 2.3 mm were cut out of the fully cured film.

Materials with four different compositions were investigated. Two of them contained a 70 wt% and two contained an 80 wt% of CIP. One of the two in the set was softer (a low-frequency shear storage modulus where *G*′ < 10 kPa) and another one harder (*G*′ > 30 kPa). Their designations and their exact storage moduli and thicknesses are listed in Table 1. Compositions with a 70 and 80 wt% of CIP were investigated because different types of magnetic-particle restructuring in a magnetic field can be expected for these two concentrations [49]. While for a sample with a 70 wt% of CIP, the percolation threshold for the filler is probably not reached in the zero field, for a sample with an 80 wt% of CIP, one could expect the existence of a three-dimensional magnetic-filler network already in the absence of magnetic field. As a result, the restructuring of the MAE surface could advance differently. Moreover, at higher filler concentrations, larger filler aggregates can be formed upon material curing, and higher fields are needed to saturate the structure.

It should be expected that, for the constant elastic modulus of the polymer matrix, the resulting shear modulus of our composite materials increases with the growing concentration of iron particles. To keep the shear modulus of the material approximately constant for different particle concentrations, one has to adjust the elastic properties of the matrix. Obviously, the matrix should be softer for a larger particle concentration. Therefore, we needed four different compositions of the PDMS matrix. For the samples with the same concentration of filling particles, the variation in the weight proportion of the crosslinker in the MAE material was primarily used to tune the shear modulus of the specimen. If the concentration of iron particles is increased, it can be compensated by increasing the weight proportion of plasticizer in the polymer matrix.

### 2.2. Optical Microscopy

Optical microscopy imaging was performed in an episcopic configuration by using long working distance optical objectives with magnification from 5× to 40× (Nikon Optiphot2-pol, Nikon, Minato, Tokyo, Japan). An external magnetic field was applied to the samples by placing a disk-shaped NdFeB-type permanent magnet (diameter 40 mm, thickness 8 mm) underneath the nonmagnetic sample holder. The magnet was mounted on a vertical translation stage, and the magnitude of magnetic field in the sample region was varied by changing the distance between the magnet and the sample. The orientation of the applied magnetic field in the investigated central area of the sample was perpendicular to the surface. The field was altered in the range from *B* = 5 mT to *B* = 200 mT.

### 2.3. Spread Optical Reflection Measurements

**Figure 1:**
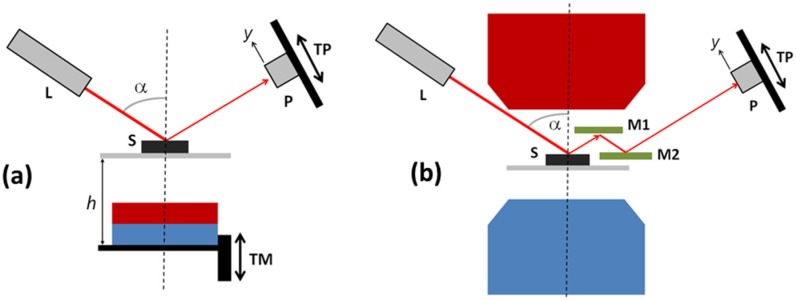
The spread optical reflectance setup (**a**) with a permanent magnet and (**b**) with an electromagnet: The red and blue elements indicate the north and south poles of the magnet, respectively. The other elements are L, laser; S, sample; P, photodiode; TM, translation stage for a magnet; TP, translation stage for a photodiode; and M1 and M2, mirrors.

Figure 1 shows two different experimental configurations that were used for the analysis of spread optical reflection. In the first configuration, the same permanent magnet as for optical microscopy imaging was used. In the second configuration, this magnet was replaced by an electromagnet (GMW model 3470) that could generate magnetic fields up to 300 mT. A He–Ne laser (JDS Uniphase) with a wavelength of 633 nm and output power of 5 mW was used as a light source. The laser beam was impinging on the sample at an incident angle *α* of approx. 55°. The diameter of the laser beam on the sample surface was approx. 1 mm.

For the qualitative observations, the reflected laser light was sent to the opaque screen placed at *d* approx. 30 cm behind the sample, and the reflection “spot” was imaged by a video camera. For the quantitative analysis, the cross-sectional profile of the spot was measured by a photodiode that was mounted on the translation stage and translated through the reflection spot. This method was selected because the dynamic range of photodiodes is much larger than the dynamic range of charge-coupled device (CCD) image sensors in conventional video cameras. The diameter of a pinhole placed in front of the photosensitive area was set to be larger than the coherence area of a specular pattern of reflected light. Consequently, the reflected intensity *I*_r_ as a function of the detector position *y* (see Figure 1) averaged over several coherence areas was detected. The obtained intensity profile of the reflection spot *I_r_*(*y*) was fitted to the Gaussian function *I*_r_(*y*) = *I*_0_⋅exp(−*y*^2^/2*σ*^2^)/(2π*σ*^2^)^1/2^, and the obtained FWHM ≈ 2.355*σ* of the fitting curve was used to calculate the effective conical spreading angle Δ*θ* = FWHM/2*d* of reflected light with respect to the specular reflection direction. Δ*θ* is proportional to the effective dispersion angle of the surface slopes on the sample.

## 3. Results

### 3.1. Optical Microscopy

**Figure 2:**
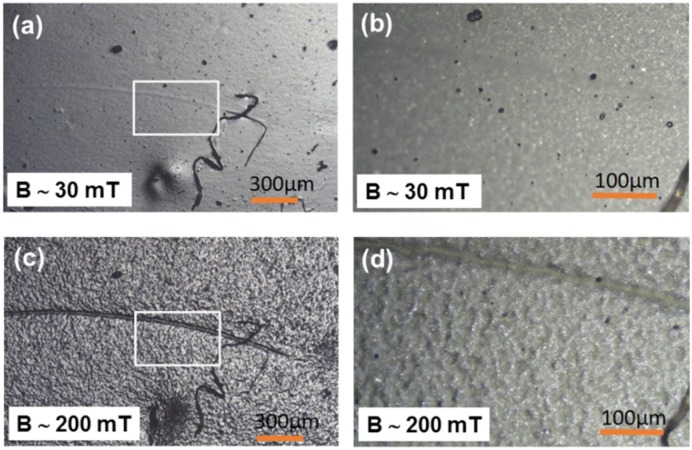
Optical microscopy images of the surface topography of soft MAE with a 70 wt% CIP: (**a**) *B* = 30 mT, magnification 5×, (**b**) *B* = 30 mT, magnification 20×, (**c**) *B* = 200 mT, magnification 5×, and (**d**) *B* = 200 mT, magnification 20×.

Optical microscopy images of a soft MAE with a 70 wt% of CIP are shown in Figure 2. A region exhibiting some unique surface imperfection was selected for imaging in order to be able to allocate one and the same surface region on various images. The upper images were observed at *B* of approximately 30 mT and the lower images at *B* of approximately 200 mT. The images on the right side correspond to magnified areas designated by white squares in the images on the left side. Both magnifications clearly reveal a strong magnetic-field-induced increase of the surface roughness that takes place in the form of “hills separated by valleys”, as already observed in our previous work. In the images on the right side, in addition to those hills and valleys, solitary carbonyl iron particles can be resolved as well.

**Figure 3:**
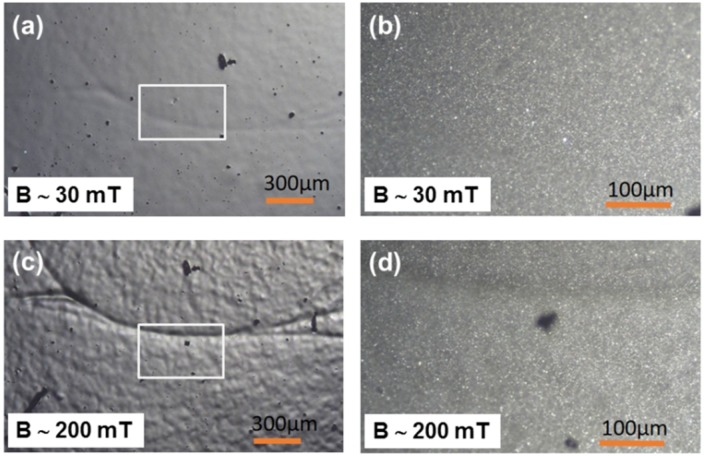
Optical microscopy images of the surface topography of hard MAE with a 70 wt% of CIP: (**a**) *B* = 30 mT, magnification 5×, (**b**) *B* = 30 mT, magnification 20×, (**c**) *B* = 200 mT, magnification 5×, and (**d**) *B* = 200 mT, magnification 20×.

Figure 3 shows an analogous set of images for the hard MAE with a 70 wt% of CIP. One can immediately notice that magnetic-field-induced surface roughening for this sample is much weaker than for the soft sample with the same filler concentration. This signifies a strong influence of the matrix softness on magnetic-field-induced surface restructuring. A similar behavior was observed also for the samples comprising an 80 wt% of CIP. Also, in this case, the softer of the two materials showed stronger field-induced roughness modifications than the harder. However, the observed modifications were, in general, smaller than for samples with a 70 wt% of CIP.

### 3.2. Spread Optical Reflection Measurements

**Figure 4:**
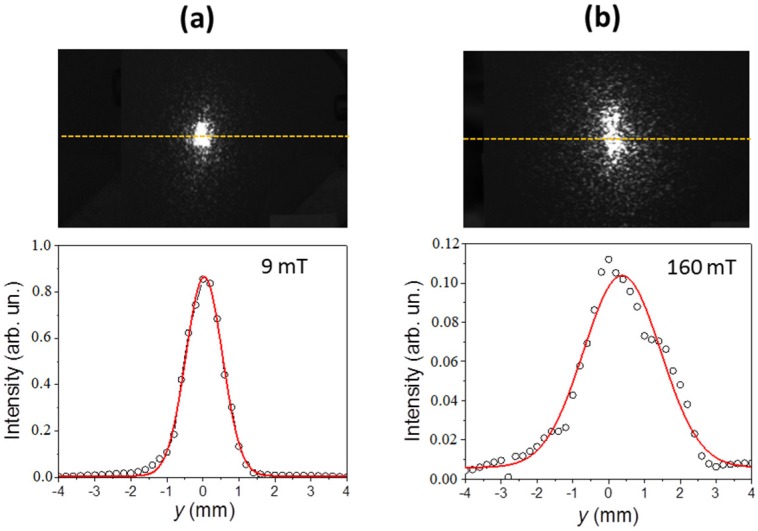
The optical reflection spot obtained by the reflection of a laser beam from the surface of a soft MAE with a 70 wt% of CIP exposed to a magnetic field of (**a**) *B* = 9 mT and (**b**) *B* = 160 mT. The upper images are the far-field reflection patterns observed on an opaque screen, and the lower images are intensity profiles measured along the yellow lines indicated in the upper images.

An examination of the spread optical reflection is a very simple and convenient tool for the in situ monitoring of field-induced surface roughness variations. In the framework of geometrical optics, the effect is attributed to a variation of the reflective surface slopes associated with an evolution of surface hills and valleys [50]. This leads to a deviation of the reflected light from the direction of specular reflection. Figure 4 shows two examples of reflection spots obtained for the soft MAE with a 70 wt% of CIP. The upper figures are video images of the spots observed on the opaque screen. The spots are elongated in the vertical direction as a result of the inclined light incidence on the observation screen. Due to the use of a coherent laser light, they exhibit a speckle pattern. One can clearly perceive that the reflection spot observed in higher magnetic field is larger in size.

The lower images in Figure 4 show the spatial dependencies of the reflected intensity *I_r_*(*y*) measured by translating the photodiode along the yellow lines in the upper images. As already mentioned, these dependencies are averaged over the speckle pattern. The red lines are fits to the Gaussian profile. Also, here, one can notice that the width of the profile obtained in the higher field is considerably larger. In addition, if comparing the values on the vertical axes, it can be noticed that the line-integrated intensity of the reflected light *I*_0_ *= ∫I_r_*(*y*)*dy* for the higher magnetic field is considerably lower than expected for the associated broadening. This suggests that the total optical power reflected from the sample decreases with an increasing field.

**Figure 5:**
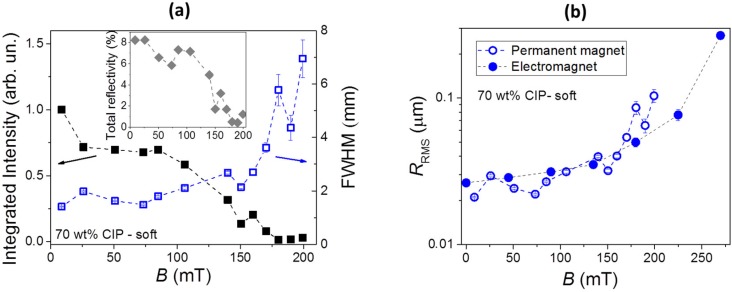
(**a**) Field dependences of the line-integrated intensity (solid symbols) and FWHM (open symbols) of the fitted Gaussian curves (see Figure 4): The inset shows the corresponding total surface reflectivity as a function of magnetic field. (**b**) The RMS surface roughness as a function of the applied magnetic field generated by a permanent magnet (open symbols) and by an electromagnet (solid symbols): The dashed lines are guides for the eye. All data are given for the soft MAE sample with 70 wt% of CIP.

Figure 5a shows the dependencies of the fitting parameters *I*_0_ and FWHM on applied magnetic field *B*. Additionally, the inset shows the corresponding total reflectivity of the sample surface, which is proportional to the product of the integrated intensity *I*_0_ and FWHM. The reflectivity was calibrated by measuring the ratio between the total reflected optical power and the incident optical power in the absence of magnetic field when the reflection is practically specular, and the value of around 8% was found. One can notice that, for a magnetic field increasing from *B* ≈ 0 to *B* = 200 mT, the total reflectivity reduces by almost a factor of 10. This means that the surface becomes considerably “darker” than in the absence of magnetic field.

As was explained previously [34], if the characteristic lateral dimension and shape of the “hills and valleys” are known, the obtained FWHM (Full Width at Half Maximum) data can be used to determine the RMS (Root Mean Square) surface roughness *R*_RMS_ of the material. In our previous investigation, we found that, by considering a triangularly shaped surface topography with a lateral size of 100 μm, a very good agreement between the *R*_RMS_ values determined from the spread reflection experiments and those deduced from the 3-D optical profilometry measurements was obtained. Therefore, also in this work, we calculate *R*_RMS_ by considering the same shape and lateral size of the surface formations as in our previous study. The corresponding result is shown in Figure 5b. The surface roughness increases from *R*_RMS_ < 20 nm at *B* = 0 to *R*_RMS_ approx. 300 nm at *B* = 270 mT. Two sets of data are shown. The first presented by open symbols corresponds to the measurements obtained by using a permanent magnet, and the second one presented by solid symbols corresponds to the measurements obtained by using an electromagnet. Both results are very similar, which indicates that the magnetic field gradients, which are much stronger in the vicinity of a permanent magnet than in between the poles of an electromagnet, do not play a vital role in field-induced roughness modifications. The observed modifications (Δ*R*_RMS_/Δ*B*) of approx. 1 μm are very similar as reported for a similar material investigated in our previous work [34], which confirms a good reproducibility of the investigated properties.

**Figure 6:**
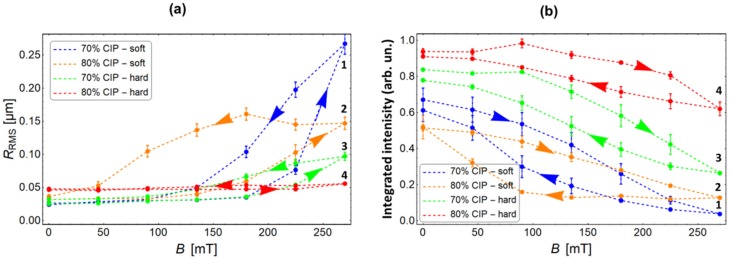
Field dependences of (**a**) the RMS roughness and (**b**) the integrated intensity for four different MAE materials: (blue, 1) soft MAE with a 70 wt% of CIP, (yellow, 2) soft MAE with an 80 wt% of CIP, (green, 3) hard MAE with a 70 wt% of CIP, and (red, 4) hard MAE with an 80 wt% of CIP. The dashed lines are guides to the eye. The arrows pointing to the right indicate data obtained during an increasing magnetic field, and the arrows pointing to the left are data obtained during a decreasing magnetic field.

All further experiments on the spread optical reflection were performed with the electromagnet. The results obtained for four different MAE materials listed in Table 1 are presented in Figure 6. The dependencies obtained during increasing and decreasing magnetic fields are shown. A profound hysteresis can be noticed for all samples. A largest surface roughness modification is observed for the soft MAE with a 70 wt% of CIP. The second largest change is detected for the soft MAE with an 80 wt% of CIP. The *R*_RMS_ of a hard MAE with a 70 wt% of CIP changes relatively little, while the *R*_RMS_ of a hard MAE with an 80 wt% of CIP remains practically constant. Similar characteristics are observed also for the integrated reflected intensity. Also, here, the largest modification is observed for the soft MAE with a 70 wt% of CIP, and the smallest is observed for the hard MAE with an 80 wt% of CIP.

**Figure 7:**
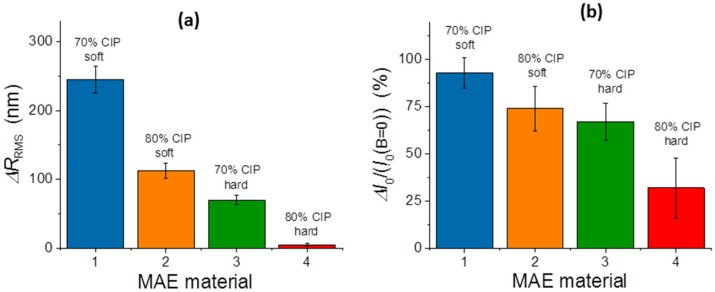
The modification of (**a**) the RMS surface roughness and (**b**) the relative modification of the reflected light intensity for different MAE materials.

A quantitative comparison between different materials is presented in Figure 7. The materials are numbered in accordance with the Table 1. Figure 7a shows the absolute modifications of the surface roughness calculated as Δ*R*_RMS_ = *R*_RMS_ (*B* = 270 mT) − *R*_RMS_ (*B* = 0), and Figure 7b gives the relative modifications of the integrated reflected intensity calculated as Δ*I*_0_/*I*_0_ = (*I*_0_(*B* = 270 mT) − *I*_0_(*B* = 0))/*I*_0_(*B* = 0). The abovementioned strong dependence of magnetic-field-induced surface modifications on bulk materials properties is, in both cases, very convincing. It can also be noticed that material softness is more important for an efficient surface restructuring than the concentration of the filler.

### 3.3. Response Processes

**Figure 8:**
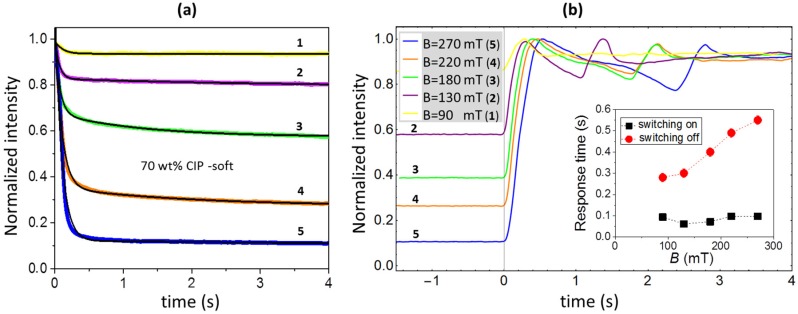
The time dependencies of the normalized reflected intensity detected after switching the magnetic field of various magnitude either (**a**) on or (**b**) off: Yellow (1) *B* = 90 mT, purple (2) *B* = 130 mT, green (3) *B* = 180 mT, orange (4) *B* = 220 mT and blue (5) *B* = 270 mT. The black lines in Figure 8a show exponential fits. The inset in Figure 8b shows the obtained response times as a function of the magnetic field. All data are given for the soft MAE with a 70 wt% of CIP.

An important question from the perspective of potential applications is how fast surface restructuring of MAE can respond to sudden changes of an applied magnetic field. To investigate this problem, we measured the time dependence of the intensity of reflected light *I_r_*_0_(*t*) in the centre of reflection spot (at position *y* = 0) after turning on and off the electromagnet. A soft MAE with a 70 wt% of CIP was probed. The results are shown in Figure 8. The magnetic field was switched on or off at *t* = 0. The inherent response time of electromagnet itself is around 0.002 s.

The normalized intensities *I*_r0_(*t*)/*I*_r0_(*t* = 0) measured after switching on the field are shown in Figure 8a. They monotonously decrease with increasing time. A relatively fast initial switching process is accompanied by a much slower and weaker relaxation effect. In accordance with the results shown in Figure 5 and Figure 6, the total intensity modification (*I*_r0_(*t* = 0) − *I*_r0_(*t* = 4 s)) increases with an increasing field magnitude *B*. The obtained dependences were fitted to a sum of two exponential-decay functions, and the corresponding fitting curves are given as black lines. The characteristic time of the faster process is associated with the characteristic switching-on time *τ*_on_ of surface restructuring.

The intensities measured after turning off the magnetic field are presented in Figure 8b and show a non-monotonic behaviour that resembles the properties of a slip-stick motion [51]. Due to a complex behaviour, no fitting procedure was performed for the obtained curves, but the characteristic switching-off time *τ*_off_ was determined as the time interval between switching off the field (*t* = 0) and the first maximum appearing in the dependence of *I*_r0_(*t*). Also, in this case, the total observed modification depends on the initial field magnitude *B*. The obtained response times as a function of *B* are presented in the inset of Figure 8b. The values of *τ*_on_ are in the range of 0.06–0.1 s and do not show any clear trend. The values of *τ*_off_ are significantly longer and increase with an increasing value of *B*.

## 4. Discussion

### 4.1. Proposed Scenario of Surface Restructuring

**Figure 9:**
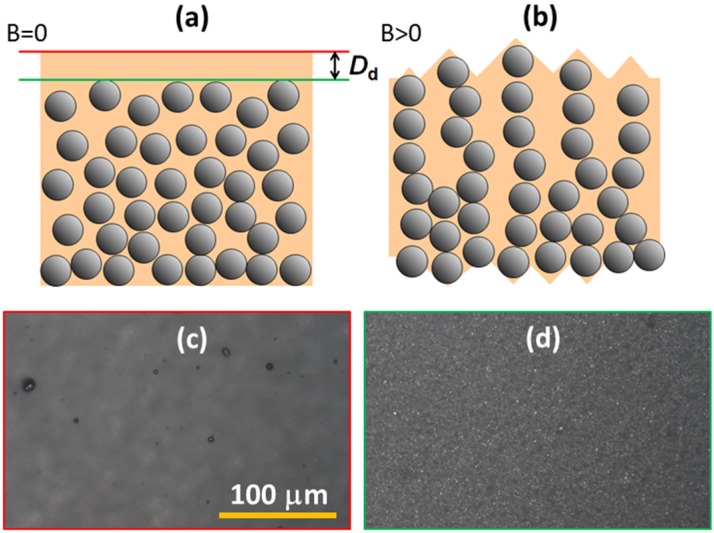
A schematic drawing of the MAE structure in (**a**) the absence of magnetic field and (**b**) in the applied magnetic field oriented perpendicular to the surface. (**c**) An optical microscopy image of the air-MAE surface with some trapped air bubbles and (**d**) of the underneath image plane located about 20 μm below the air-MAE interface, in which single carbonyl iron microparticles can be seen.

In the absence of a magnetic field, all MAE composites investigated in our study exhibit an RMS roughness below 50 nm, which is very much below the microparticle size (diameter of 4.5 μm). Such a low surface roughness is explained by the existence of a topmost polymeric surface layer that is depleted of microparticles, as depicted in Figure 9a. This layer is most probably formed during the degassing phase of the liquid prepolymer mixture, as a consequence of surface tension and gravitational sedimentation. The sedimentation of microparticles is a known challenge in magnetorheological fluids [52]. In MAEs, this sedimentation is prevented after curing, and it is difficult to observe it directly in such thin samples. Previous X-ray micro-computed tomography measurements on nominally isotropic MAE samples also indicated a homogeneous particle number density within the sample with strong deviations at top and down surfaces [53].

When an external magnetic field in the direction perpendicular to the surface is applied, two processes take place as indicated in Figure 9b. The first is the development of chains of microparticles, which results in the formation of surface valleys and hills. The second is thinning and possibly a complete vanishing of the depletion layer due to an expansion of the particle chains towards the surface. The first process induces the observed spreading of optical reflection, and the second causes a decrease in the total reflected optical power.

The thickness of the depletion layer *D*_d_ can be deduced by examining the image sharpness of different surface details during the relative motion of the high magnification microscopy objective (40×, NA 0.5) with respect to the sample. Figure 9c shows an image obtained by focusing the objective precisely at the MAE-air interface (red line in Figure 9a), where some residual trapped air bubbles can be noticed. Figure 9d shows another image that is typically revealed about 20 μm below the MAE-air interface (green line in Figure 9b) and on which single carbonyl iron microparticles can be clearly resolved. Both images were obtained in the absence of a magnetic field. When a magnetic field with growing magnitude is applied to the sample, the two characteristic image planes at first become more and more indistinct and then they merge into one and the same relatively hazy image.

### 4.2. Main Observations

Our results show that the magnetic-field-induced modification of the surface roughness of MAE strongly depends on the material composition.

Particle concentration is generally known to be the parameter that has a very strong influence on the properties of MAE [54]. Our measurements reveal that, when comparing materials with the same elastic modulus, the material with a 70 wt% of CIP shows much larger field-induced surface restructuring than the material with an 80 wt% of CIP. This is attributed to the mutual hindrance of particle rearrangement in the sample with a larger particle concentration. If particles cannot move to form the chain-like structures, then the associated surface mountains also do not form. Romeis et al. demonstrated theoretically that the formation of elongated structures within a MAE material may become impossible for high concentrations of magnetic particles due to purely geometric constraints [55].

The largest changes of surface roughness and optical reflectance were detected for the soft MAE with a 70 wt% of CIP, and the second largest change was detected for the soft MAE with an 80 wt% of CIP. This is in agreement with recent molecular dynamics simulations of MAE thin films, which resolved a strong correlation between the matrix softness and surface restructuring [33]. Softer matrices enable a larger extension of the microparticle chains along the direction of an applied magnetic field, which consequently leads to the formation of well-separated mountains that are pushed out from the surface by the underneath chains [37]. Cvek et al., who have recently investigated the effect of microparticle-grafting on various MAE features, also found out that field-induced roughness modifications are very strongly correlated with the value of the elastic modulus of the medium [35].

For all four MAE compositions investigated in our work, we observed a profound hysteresis of the roughness modifications taking place during increasing and decreasing magnetic fields. Despite this hysteresis, when the magnetic field was set back to *B* = 0, the initial surface roughness was reestablished. In one of the experiments, this effect was tested by applying the field up to 430 mT, and nevertheless, a reversible behavior was found.

Another interesting observation is the existence of an elastomeric surface layer that practically does not contain any microparticles. This explains why the surface roughness in a zero magnetic field is much smaller than the typical size of microparticles. The obtained values of RMS surface roughness at *B* = 0 lie in between 20–50 nm. However, the real values are very probably even lower because, in our analysis of the spread optical reflection, we neglected the intrinsic width of the incident optical beam. Surfaces that are very flat in the absence of a magnetic field and that became quite rough when the field was applied, are very interesting for applications in magnetically tunable adhesion and friction coatings [39,40].

Due to the presence of the abovementioned microparticle depletion layer, a substantial part of the optical power is reflected from the air-PDMS interface in the absence of magnetic field, leading to a relatively large surface reflectivity. With an increasing magnetic field, the microparticles are shifted towards the surface, and the thickness of the depletion layer is reduced. Consequently, the absorption and scattering of light by microparticles is becoming more and more important, which leads to an increased optical losses and, therefore, to a reduced reflectivity.

### 4.3. Remaining Open Questions

All results reported in this work were obtained by investigating the upper surface of MAE samples, i.e., the surface that was in contact with air during the curing process. However, the lower surface, that is in contact with the material of the petri dish (polystyrene), might have different features. Our preliminary results show that, even without a magnetic field, this surface is quite rough and far less reflective than the upper surface. Nevertheless, it still exhibits some magnetic-field-induced modifications. Further investigations are in progress to resolve the nature and the origin of the observed differences.

Further investigations are needed also to clarify the unusual time response of the reflected light to switching off the magnetic field. The oscillations shown in Figure 8 might be associated with roughness modifications, but they might also be related to the slip-stick motion of the entire sample. Therefore, the sample mount should be further improved to minimize the latter effects, and a systematic analysis of the correlation between surface roughness modifications and slip-stick movement has to be performed.

## 5. Conclusions

Our observations demonstrate that there is still a lot of room for the further optimization of the MAE composition in order to achieve the best possible tunability of surface roughness with an applied magnetic field. As it follows from our analysis, large matrix softness is definitely beneficial, but care needs to be taken to assure the necessary stability and durability of the material. Besides this, also the entire material processing protocol needs to be further optimized in order to obtain surface structures (depletion layer thickness, shape of surface hills generated, their lateral size, etc.) that can best facilitate the desirable type of restructuring.

## Figures and Tables

**Table 1:** A list of the investigated materials and their properties.

Material	Designation	*G*′ (kPa)	Thickness (mm)
1	70% CIP; soft	9.1 ± 0.1	2.28 ± 0.01
2	70% CIP; hard	32.3 ± 0.4	2.24 ± 0.01
3	80% CIP; soft	8.0 ± 0.1	2.35 ± 0.01
4	80% CIP; hard	35.8 ± 5.3	2.48 ± 0.01
